# The First Report of Rhino DNA in Thailand: A Possible Extinct Indian Javan Subspecies, *Rhinoceros sondaicus inermis*

**DOI:** 10.3390/ani15121678

**Published:** 2025-06-06

**Authors:** Yada Katanyuphan, Pipad Krajaejun, Athiwat Wattanapituksakul, Wunrada Surat

**Affiliations:** 1 Department of Genetics, Faculty of Science, Kasetsart University, Bangkok 10900, Thailand; yadoze@gmail.com; 2 Department of History, Faculty of Liberal Arts, Thammasat University, Pathum Thani 12120, Thailand; pipad_k@yahoo.com; 3 The Timing and the Cause of the Transition in Domestic Cattle Species in Thailand Project, Department of Genetics, Faculty of Science, Kasetsart University, Bangkok 10900, Thailand; essathiwat@gmail.com

**Keywords:** Javan rhino, D-loop, ancient DNA, extinction, Thailand, subspecies

## Abstract

The Javan rhino is a critically endangered species that has become extinct in several Asian countries, including Thailand. There are three Javan rhino subspecies: *Rhinoceros sondaicus sondaicus*, *R. s. annamiticus*, and *R. s. inermis*, and the first two subspecies existed in Thailand decades ago. However, no genetic study on Thai rhinos has been reported. In this study, we amplified a partial D-loop region of two ancient rhino specimens dated to approximately 100 years before present, from southwest Thailand. Then, we constructed phylogenetic trees and a haplotype network based on the D-loop sequences of the Thai rhinos and 39 Javan, Sumatran, and Indian rhinos from the GenBank database. The ancient Thai rhino DNA belonged to Javan rhinos and was clearly separated from *R. s. annamiticus* and *R. s. sondaicus*. The nucleotide differences among the ancient Thai, *R. s. annamiticus*, and *R. s. sondaicus* rhino DNA were 5.14–8.30%, similar to those between two white rhino subspecies. Moreover, species delimitation analyses confirmed that the ancient Thai rhinos should belong to another Javan rhino subspecies, possibly the extinct Indian Javan rhino. This is the first report of rhino DNA from Thailand and possibly the Indian Javan rhino DNA, which could be used for evolutionary studies.

## Simple Summary

The Javan rhino is a critically endangered species where its wild population, approximately 50 individual rhinos, survive only in Java, Indonesia. In the past, there were three Javan rhino subspecies: the Indonesian Javan rhino, the Vietnamese Javan rhino, and the Indian Javan rhino, of which only the first subspecies still exists. The last Vietnamese Javan rhino was killed in 2010, while the Indian Javan rhino became extinct in the early nineteenth century. The Vietnamese Javan and the Indonesian Javan rhinos had been reported to exist in Thailand. In this study, two rhino remains, dated to approximately 100 years before present and found in Prachuap Kirikhan, the west of Thailand, had their species identified based on a partial DNA sequence. The results of genetic analyses demonstrated that the Thai rhino remains belonged to Javan rhinos. However, they had dramatic genetic distinctions from both the Indonesian and the Vietnamese Javan subspecies and possibly belonged to the Indian Javan rhino. The findings suggest that the Indian Javan existed in Thailand at least 100 years ago and that this Javan rhino subspecies had a broader habitat than previously believed. Our new DNA sequences can be used for conservation and evolutionary studies of this rhino species.

## 1. Introduction

Rhinoceroses, typically referred to as rhinos, are in the family Rhinocerotidae, which comprises more than 100 species. While they were historically distributed in North America, Africa, Europe, and Asia, they became extinct in North America and Europe approximately 4 million and 12,000 years ago, respectively [1,2]. Today, there are only five extant rhino species: *Rhinoceros sondaicus* (the Javan rhino), *Dicerorhinus sumatrensis* (the Sumatran rhino), *Rhinoceros unicornis* (the Indian rhino), *Ceratotherium simum* (the white rhino), and *Diceros bicornis* (the black rhino), which are all in danger of extinction [2]. Rhino poaching remains the most significant cause of rhino population decline. In Africa, rhino poaching has increased by 4% from 2022 to 2023 [3]. In addition, 26 rhinos were killed in Ujung Kulon National Park, Indonesia, from 2019 to 2023 [3]. The Javan, Sumatran, and black rhinos are critically endangered species, whereas the Indian and white rhinos are vulnerable and near-threatened species, respectively [3]. The Javan, Sumatran, and Indian rhinos are native to South and/or Southeast Asia, while the white and black rhinos are native to Africa [2].

The Javan rhino is the second-rarest rhino species, with approximately 50 reported individual rhinos [3]. Historically, this rhino species was distributed in India, Bangladesh, China, Cambodia, Myanmar, Thailand, Laos, Vietnam, Malaysia, and Indonesia. However, it is now only found in Ujung Kulon National Park in southwest Java, Indonesia [3,4]. This species comprises three subspecies—*R. s. sondaicus* (the Indonesian Javan rhino), *R. s. inermis* (the Indian Javan rhino), and *R. s. annamiticus* (the Vietnamese Javan rhino)—although only the first is an extant subspecies [5,6]. The Indonesian Javan rhino is found in Thailand, Malaysia, and the islands of Java and Sumatra in Indonesia [7]. The Vietnamese Javan rhino was distributed in Laos, Cambodia, Eastern Thailand, and Vietnam but became extinct after the last rhino was killed in Cat Tien National Park in Vietnam in 2010 [6,8]. The Indian Javan rhino was distributed in Northeast India, Bangladesh, and Myanmar but became extinct in the early nineteenth century [8,9].

The Javan and Sumatran rhinos were found in Thailand and have been reserved as wild animals since 1960 [10]. In addition, Indian rhino fossils have been found in West and Northeast Thailand [11,12]. Moreover, a fossil of the skull and mandible of *Aceratherium porpani*, a new Late Miocene species of hornless rhinos, was found in Northeast Thailand [13]. Therefore, at least four rhino species have historically lived in Thailand. However, no rhino species is now found in Thailand, and the last rhino traces were found in the Hala-Bala Wild Sanctuary in 1997 [14]. Moreover, no genetic data on Thai rhinos have been reported. The GenBank database contains genetic data on Javan rhinos only from Vietnam and Indonesia, with only a few DNA sequences of these subspecies recorded. Additional genetic data on the Javan rhino could shed light on the evolution of this rhino species.

In this study, we aimed to identify the species and subspecies of the ancient rhino specimens found in Thailand, using the partial D-loop region to construct phylogenetic trees and a haplotype network. This study is the first to report genetic data from Thai Javan rhinos. Its results can be used for conservation efforts and to study the evolution of the critically endangered Javan rhino.

## 2. Materials and Methods

### 2.1. Sample Preparation and DNA Extraction

**Figure 1:**
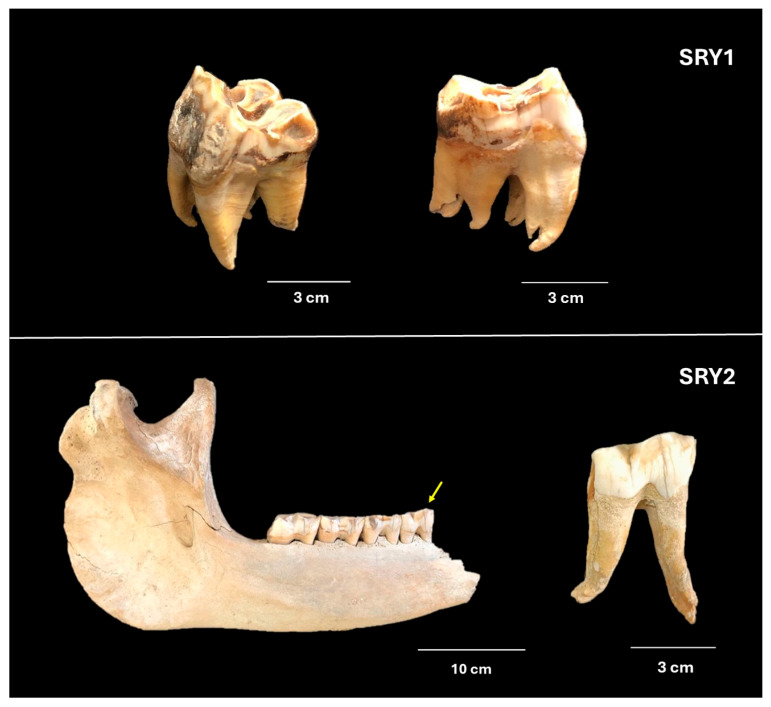
Two ancient rhino specimens (SRY1 and SRY2) from the rhino skeleton collection room in the tourist service centre, Khao Sam Roi Yot National Park, Prachuap Khiri Khan Province, Thailand, dated to approximately 100 years before present. The yellow arrow points to the tooth that was used in this study.

Two rhino teeth were collected from the rhino skeleton collection room in the tourist service centre, Khao Sam Roi Yot National Park, Prachuap Khiri Khan Province, Thailand, dated to approximately 100 years before present (YBP). Two teeth from different individual rhinos were examined: one was already unattached from the mandible (SRY1), and the other was extracted from the mandible (SRY2; Figure 1). The specimens were surface cleaned using a dental drill, and each side of them was exposed to UV for 30 min in a laminar hood. Next, the cleaned specimens were ground into fine powder using a sterilised pestle and mortar. Then, DNA was extracted from approximately 0.25 mg of the powder following the protocol described by Damgaard et al. (2015) [15]. Blank extracts were performed alongside the ancient specimens.

### 2.2. Amplification of the Partial D-Loop Region

A 300 bp D-loop region was amplified by nested PCR. Three sets of primers were used [16]: Primer I (RDL-F1: 5′-TGCATTAAATTGTWTGCCCCATGC-3′, RDL-R1: 5′-GGCCCGATCAATAATAHAATGTACTATGC-3′), Primer II (RDL-F2R: 5′-GAGGAGATATTACATAAGACATYAGG-3′, RDL-R2: 5′-GTTGWCTAGAAATGATTTGACTTG-3′), and Primer III (RDL-F3: 5′-GGCCGCATAGTACATTHTATTATTGATCG-3′, RDL-R3: 5′-ATGGGCCCGGAGCGAGAAC-3′). The forward primer of Primer I and the reverse primer of Primer III were used for the first round of the nested PCR, and then each primer set was used in the second round of PCR. The 50 µL PCR mixture consisted of 5 µL of 10× PCR buffer, 1.5 µL of 50 mM MgCl_2_, 0.2 µL of Platinum^TM^ *Taq* polymerase (Invitrogen, Carlsbad, CA, USA), 0.5 µL of each primer (10 µM), 0.5 µL of bovine serum albumin, 1 µL of 10 mM dNTP mixture, 4 µL of DNA template, and distilled water. A PCR negative control and blank extract were included in all sets of reactions. The PCR conditions for the first round of PCR were an initial denaturation at 94 °C for 1 min; 40 cycles of denaturation at 94 °C for 30 s, annealing at 54 °C for 30 s, and extension at 72 °C for 1 min; and a final extension at 72 °C for 2 min. The PCR conditions for the second round of PCR were according to Katanyuphan and Surat (2023) [16]. Then, the sizes of the PCR products were visualised via 2% agarose gel electrophoresis and Novel Juice staining (Bio-Helix, Taiwan). The PCR products were sequenced using Sanger sequencing using the ABI 3730XL DNA Analyzer (Applied Biosystems™, Foster City, CA, USA).

### 2.3. Sequence Processing

Raw sequencing data from the forward and reverse primers were carefully verified for the existence of ambiguous chromatograms before being assembled using BioEdit v7.2.5 [17], and the partial D-loop sequences amplified from three sets of primers were overlapped into a final length of 257 bp. Subsequently, nucleotide sequences obtained herein were compared to sequences in the GenBank database through the BLASTN search for primarily species identification.

Prior to further sequence analyses, the partial D-loop sequences obtained from rhinos in the present study (*n* = 2) were aligned with those partial D-loop and 39 mitochondrial DNA sequences from Javan (*n* = 6), Sumatran (*n* = 16), and Indian (*n* = 17) rhinos sourced from the GenBank database, employing the Clustal Omega ClustalW Multiple Alignment version 2024 (https://www.ebi.ac.uk/jdispatcher/msa/clustalo; accessed on 26 November 2024) [18]. Then, the sequences in the current study and those from GenBank were trimmed to a length of 253 bp using BioEdit v7.2.5.

### 2.4. Analyses of Phylogeny, Haplotype Network, and Nucleotide Polymorphism

To identify the species of the ancient rhino specimens, the partial D-loop sequences of the ancient rhinos and the three Asian rhinos from GenBank database were used to construct phylogenetic trees using the neighbour-joining (NJ) method and maximum likelihood (ML) method with MEGA (version 11) and IQ-TREE (version 1.6.12) running for 1000 replicates [19,20]. The best-fitting evolutionary models were predicted by ModelFinder [21], and the Kimura-2 parameter and the HKY + F + G4 models were selected for the NJ and ML phylogenetic analyses, respectively. Subsequently, the polymorphic sites within the Javan rhinos were detected using DnaSP v6.12.03 [22]. To visualise the genetic relationship among rhinos from Thailand and Asian rhinos from other countries, the partial D-loop sequences of two rhino specimens in the present study and the 39 Asian rhino sequences were used to construct a haplotype network by PopART [23] with the TCS algorithm [24].

### 2.5. Species Delimitation Analyses

To determine the boundary among the ancient Thai rhinos and other Javan subspecies, nine methods of species delimitation were conducted on the partial D-loop sequences of five rhino species, including eight subspecies, from the GenBank database; *Rhinoceros sondaicus* (*R. s. annamiticus*, *n* = 1 and *R. s. sondaicus*, *n* = 5) [4,25,26], *Rhinoceros unicornis* (*n* = 17) [27,28], *Dicerorhinus sumatrensis sumatrensis* (*D. s. sumatrensis*, *n* = 11 and *D. s. harrissoni*, *n* = 5) [4,26,29], *Ceratotherium simum* (*C. s. simum*, *n* = 8 and *C. s. cottoni*, *n* = 2) [25,30,31], and *Diceros bicornis michaeli* (*D. b. michaeli*, *n* = 5 and *D. b. minor*, *n* = 8) [25,32,33,34,35] were included in the analysis (Table S1). Two genetic distance-based methods—assemble species by automatic partitioning (ASAP) [36] and automatic barcode gap discovery (ABGD) [37]—were performed. ABGD and ASAP delimitations were conducted using three substitution models (simple-distance [p-distance], JC69 [38], and K80 [39]) on the ABGD (https://bioinfo.mnhn.fr/abi/public/abgd/abgdweb.html; accessed on 17 January 2025) and ASAP web servers (https://bioinfo.mnhn.fr/abi/public/ asap/asapweb.html; accessed on 17 January 2025), respectively. In addition, 10 replicates were run for each analysis to assess the consistency of the results.

Additionally, the phylogeny-based methods, consisting of the single-rate (sPTP), multiple-rate (mPTP), and Bayesian (bPTP) Poisson Tree Processes, were implemented using web servers (https://mptp.h-its.org/#/tree and https://species.h-its.org/; accessed on 18 January 2025) [40,41]. The previous ML tree from the phylogenetic analysis was used as an input file for the sPTP and mPTP analyses. For the bPTP method, the Bayesian Inference (BI) phylogenetic tree is required. Therefore, the BI analysis was implemented using BEAST v2.4.1 [42], and the best-fitting evolutionary model was selected by bModelTest [43]. The strict clock model was applied with a Yule model tree prior. The analysis was run for two independent chains, and the 10,000,000 MCMC steps were run for each analysis until convergence. Subsequently, the tree was annotated using TreeAnnotator v2.7.7 [42] and imported into the bPTP analysis.

## 3. Results and Discussion

### 3.1. Successful Amplification and Species Identification

The PCR product of SRY2 could be detected from the first round of PCR, while the PCR product of SRY1 could be amplified by nested PCR. This finding suggested that the DNA might be more damaged in SRY1 than in SRY2. While the ages of these two specimens were estimated to be similar, their conditions differed (Figure 1). SRY2 is an intact tooth, which could preserve DNA well, whereas SRY1 is a broken tooth, which could be penetrated easily by water and microbes, damaging or destroying the DNA [44].

**Figure 2:**
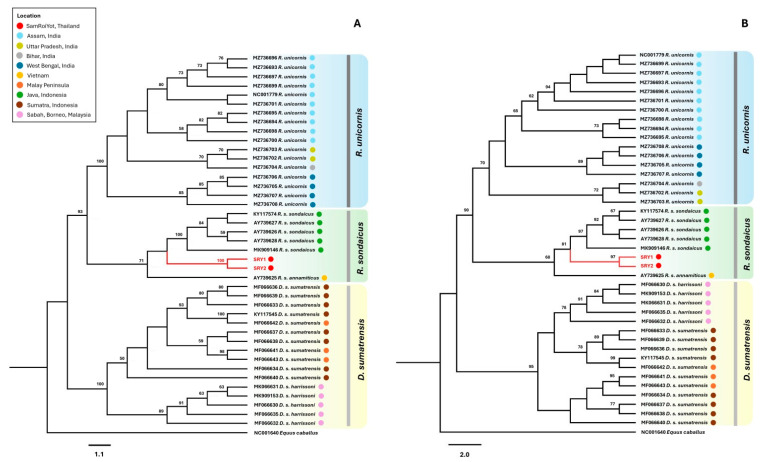
Neighbour-joining (**A**) and maximum likelihood (**B**) phylogenetic trees reconstructed from the 253 bp D-loop sequences of two ancient Thai specimens in this study and the Javan (*R. sondaicus*), Sumatran (*D. sumatrensis*), and Indian rhinos (*R. unicornis*) from the GenBank database. Equus caballus (NC001640) was included as the outgroup. The numbers at the nodes indicate frequency values above 50% with 1000 replications. The scale bar represents the number of nucleotide substitutions per site.

The 257 bp D-loop sequences were used to preliminarily identify the species using BLAST+ 2.16.0. SRY1 and SRY2 were identified as Javan rhinos, showing the greatest similarity to the Vietnamese Javan subspecies (*R. s. annamiticus*) with 94.07% and 94.47% identity, respectively. In addition, the phylogenetic tree confirmed that SRY1 and SRY2 belonged to Javan rhinos, showing a very close relationship to each other with 100% and 97% bootstrap values in the NJ and ML trees, respectively (Figure 2). The Indonesian Javan samples were clustered together with 100% and 97% bootstrap values in the NJ and ML trees, respectively, while the Vietnamese Javan was separated from other Javan samples with bootstrap values of 71% and 68% in the NJ and ML trees, respectively. Interestingly, SRY1 and SRY2 were separated from both the Indonesian (*R. s. sondaicus*) and Vietnamese (*R. s. annamiticus*) Javan rhinos with 81% and 68% bootstrap values on the divergence nodes (Figure 2B). Two Sumatran rhino subspecies, *D. s. sumatrensis* and *D. s. harrissoni,* were separated into different clades with 100% and 95% bootstrap values in the NJ and ML trees, respectively. While there are no subspecies of Indian rhino, this species can be divided into three evolutionary units: West Bengal, Assam, and Uttar Pradesh [27]. Indeed, the phylogenetic trees also separated these samples into three subclades with bootstrap values of >50%. These results show that the D-loop region has sufficient nucleotide diversity to separate the evolutionary units and subspecies of these rhino species.

### 3.2. Nucleotide Diversity, Haplotype Network Analysis, and Species Delimitation

The analysis of nucleotide diversity revealed that the number of unique nucleotide positions was highest among the ancient Thai samples (SRY1 and SRY2; *n* = 9), followed by the Indonesian (*n* = 7) and Vietnamese (*n* = 5) Javan rhino samples, which were all transitions (Table 1). When examining the relationships of the ancient Thai samples with the Vietnamese and Indonesian Javan rhino samples, it was found that they were closer to the Vietnamese Javan rhino (15–16 nucleotide differences, 5.33–6.32%) than the Indonesian Javan rhino samples (17–21 nucleotides, 6.72–8.30%). The Indonesian and Vietnamese Javan rhino samples were categorised into different subspecies, showing 13–17 nucleotide differences (5.14–6.72%). These results show that the nucleotide differences between the Indonesian and Vietnamese Javan rhino samples are similar to those between the ancient Thai samples and the Vietnamese and Indonesian Javan rhino samples.

**Figure 3:**
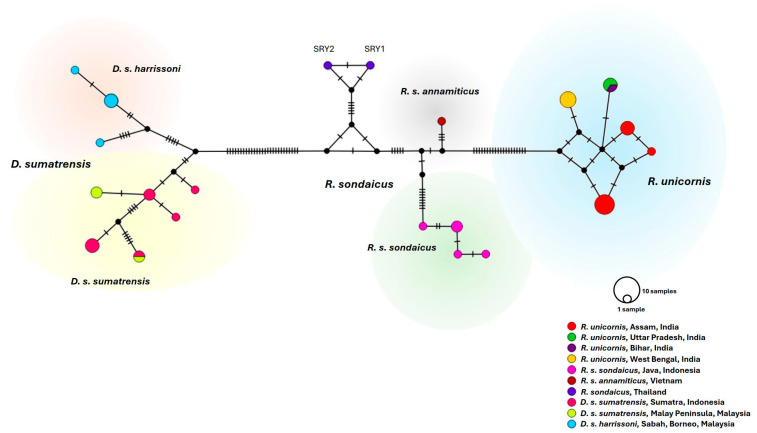
The haplotype network based on the 253 bp D-loop of the ancient Thai specimens in this study and the Javan (*R. sondaicus*), Sumatran (*D. sumatrensis*), and Indian rhinos (*R. unicornis*) from the GenBank database. The size of each circle is proportional to the number of sequences. Different rhino species from different locations are shown in different colours. The cross-lines on each branch indicate the number of mutations.

**Figure 4:**
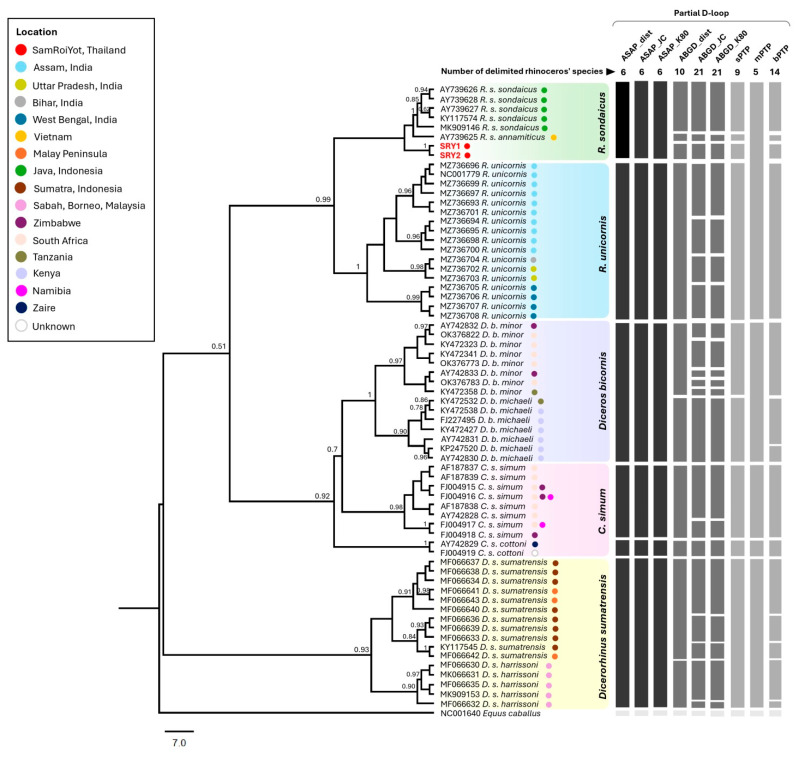
The partial D-loop was analysed by the automatic barcode gap discovery (ABGD), assembled species by automatic partitioning (ASAP), and (B) Poisson tree process (PTP) delimitation. ABGD and ASAP were conducted with three substitution models—p-distance, JC69, and K80—whereas the PTP was conducted with single-rate (sPTP), Bayesian (bPTP), and multiple-rate (mPTP) approaches. Each horizontal bar signifies a putatively delimited species, while each vertical bar represents a delimitation method. The posterior probabilities that are higher than 0.50 are shown at the nodes.

Previous analyses of partial mitochondrial DNA (mtDNA) sequences (413 bp, containing 21 bp of the 3′ end of tRNA-Pro and 392 bp of the adjacent partial D-loop region) clearly separated the Indonesian and Vietnamese subspecies of Javan rhinos into different groups with nucleotide differences of 4.8–5.1% [4,25]. The white rhino comprises two subspecies—*C. s. simum* and C. s. *cottoni*—and nucleotide differences in the partial mtDNA between them were reported as 7.2% [25,45]. The black rhino comprises three extant subspecies—*D. b. minor*, *D. b. michaeli*, and *D. b. bicornis* –and nucleotide differences in the partial mtDNA between *D. b. minor* and *D. b. michaeli* were previously reported as 3.7–4.3% [35]. The Sumatran rhino comprises two extant subspecies—*D. s. sumatrensis* and *D. s. harrissonni*—and no nucleotide differences have been reported between them [25,29,46]. However, our study found that the two Sumatran rhino subspecies differed by 18–26 nucleotides (7.11–10.28%; Figure 3). In addition, nucleotide differences among the ancient Thai, Indonesian Javan, and Vietnamese Javan samples (5.14–8.30%) were similar to those between two African white rhino subspecies but greater than those between the African black rhino subspecies. Moreover, the haplotype network showed that the Javan rhino samples from these three countries were clearly separated into different groups (Figure 3). Furthermore, the nucleotide differences within the Indonesian Javan rhino samples did not exceed seven substitutions (2.77%). Therefore, the ancient Thai Javan rhinos are clearly separated from the Javan rhino subspecies, indicating that nucleotide polymorphisms within the partial D-loop region can be used to track the origin of the Javan rhino specimens, including ancient rhino remains. In addition, the Bayesian tree showed that the Thai samples were clearly separated from the other two Javan rhino subspecies, and they were clustered together with 100% of posterior probability, indicating that they were very closely related to each other and that they should belong to the same subspecies (Figure 4). Similarly, the same subspecies within other extant rhinos—the white, black, and Sumatran rhinos—were clustered together with high posterior probabilities (90–100%). Moreover, the analyses of species delimitation showed that five of nine methods—ABGD (p-distance, JC69, and K80), sPTP, and bPTP—can separate SRY1 and SRY2 from the Vietnamese and Indonesian Javan rhinos (Figure 4). The ABGD (p-distance) can separate subspecies within all four extant rhino species, while ASAP methods cannot differentiate subspecies of almost all rhino species except the white rhinos (*C. simum*). The result has shown that the ancient Thai rhinos were neither *R. s. annamiticus* nor *R. s. sondaicus*, but they possibly belonged to a different Javan rhino subspecies.

Several reports have indicated that Thailand was the habitat for two Javan rhino subspecies: the Indonesian and Vietnamese rhinos. In contrast, the extinct Indian Javan subspecies (*R. s. inermis*) was distributed in Northeast India, Bangladesh, and Myanmar [6,7,8]. Therefore, the ancient Thai specimens may belong to one of the two subspecies. However, the nucleotide differences, haplotype network, and analysis of ASAP and PTP delimitation indicate that the ancient Thai specimens are clearly separated from the two Javan subspecies. Thus, they should be categorised into another Javan rhino subspecies, the extinct Indian Javan (*R. s. inermis*) rhino, indicating that the habitat of this Javan subspecies must be broader than previously believed. Similarly, several reports have indicated that the Indian rhino (*R. unicornis*) was only distributed in South Asian countries; however, Indian rhino fossils have been found in Western and Northeastern Thailand [11,12,27]. In addition, the ancient Thai rhino specimens used in our study were found in Prachuap Kiri Khan Province, close to the border with Myanmar, one habitat of the Indian Javan rhino [27]. Our findings show that the Indian Javan rhino possibly existed in Thailand at approximately 100 YBP. Since no DNA sequences of the Indian Javan rhino have been reported, we suggest that the Thai Javan rhinos could belong to the Indian Javan rhino subspecies. Further experimentation with more rhino specimens is needed to help us confirm whether these Thai rhino remains belong to the extinct Javan rhino or not.

The Javan rhino is one of the rarest mammals, with approximately 50 individual rhinos surviving in the wild only in Java, Indonesia [3]. In the past, the Javan rhino had been distributed throughout Southeast Asia, but it became extinct in Thailand decades ago. The oldest Javan rhino remains (dated to approximately 3000 years ago) were found at the Ban Chiang World Heritage site, Udon Thani Province, Northeast Thailand [47]. This rhino species has dramatically reduced in number since the 19th century, mainly due to poaching [4]. Recently, 26 rhinos were killed in Ujung Kulon National Park, Indonesia, from 2019 to 2023 [3]. Hence, the conservation of this rhino species is urgent, and genetic information is very crucial for its conservation plan. In addition, genetic data of the rarest species is also important for evolutionary study. Previous studies indicated that the Javan rhino has the closest relationship to the Indian rhino (*Rhinoceros unicornis*) and that they were split approximately 12–13 million years ago [48,49]. However, there is no genetic study on the examination of the divergence between the three Javan rhino subspecies. Here, we provide two DNA sequences of two Indian Javan rhinos, and there is one DNA sequence of the Vietnamese Javan in the GenBank database. In the future, further examination with more DNA sequences of the two extinct Javan rhino subspecies will gain insight into the evolution of all three Javan rhino subspecies.

## 4. Conclusions

This is the first genetic study on Thai rhino DNA. The partial D-loop sequences from the ancient Thai specimens dated approximately 100 YBP were successfully amplified, and the phylogenetic analyses confirmed that the ancient Thai rhino belonged to the Javan rhino. The haplotype network, the percentage of nucleotide differences, and the species delimitation analyses indicated that the ancient Thai rhino remains were clearly separated from the Vietnamese and the Indonesian Javan rhinos, and we suggested that they could possibly be categorised into the extinct subspecies, the Indian Javan rhino. The new DNA sequences could be used for conservation plans and evolutionary studies concerning the critically endangered Javan rhino.

## Figures and Tables

**Table 1:** The polymorphic sites within the partial D-loop sequences of the Javan rhinos (*Rhinoceros sondaicus*) from three countries: Thailand, Vietnam (*R. s. annamiticus*), and Indonesia (*R. s. sondaicus*).

Haplotype	*n*	Variable Sites																										Accession no.	Location/Population
		19	26	27	43	48	56	63	64	67	74	78	84	104	105	111	124	134	143	171	174	191	202	214	216	218	242		
H3	2	T	A	G	T	T	G	A	G	C	G	A	G	A	C	A	A	G	A	T	T	A	A	C	G	A	C	KY117574, AY739627	Java, Indonesia
H4	1	.	.	.	.	.	.	.	.	.	.	.	.	.	.	.	.	.	.	C	.	.	.	.	.	.	.	MK909146	Java, Indonesia
H6	1	C	.	.	C	.	.	.	.	.	.	.	.	.	.	.	.	.	.	.	.	.	.	.	.	.	.	AY739626	Java, Indonesia
H5	1	C	.	.	C	.	.	.	.	.	.	.	.	.	.	.	.	.	.	.	.	.	.	.	.	G	.	AY739628	Java, Indonesia
H7	1	.	G	.	.	.	A	G	.	T	A	G	A	G	.	.	G	A	G	C	.	.	G	.	C	.	.	AY739625	Vietnam
H2	1	.	.	A	.	C	A	G	A	T	.	G	A	G	T	G	.	.	.	.	C	G	G	T	.	.	T	PV089846 (SRY1)	Thailand (this study)
H1	1	.	.	A	.	C	A	G	A	T	A	G	A	G	T	G	.	.	.	.	C	G	G	T	.	.	T	PV089847 (SRY2)	Thailand (this study)

## Data Availability

The raw data supporting the conclusions of this article will be made available by the authors upon request.

## Supplementary Materials

The following supporting information can be downloaded at: https://www.mdpi.com/article/10.3390/ani15121678/s1, Table S1: The details of the partial D-loop sequences from Asian and African rhinos from the GenBank database included in this study.
