# Supplementary material for: The First Report of Rhino DNA in Thailand: A Possible Extinct Indian Javan Subspecies, Rhinoceros sondaicus inermis
Source: Animals (Basel). 2025 Jun 6;15(12):1678. doi: 10.3390/ani15121678 (PMC12189269; doi:10.3390/ani15121678)
Supplement: Supplementary file 1 [file animals-15-01678-s001.zip › animals-3622175-supplementary.pdf]

Table S1. The details of the partial D-loop sequences from Asian and African rhinos from the GenBank database included in this study.

No.	Species	Accession number	Location	Length (bp)	Reference	Note
1	*Dicerorhinus sumatrensis sumatrensis*	KY117545	Sumatra, Indonesia	1,015	Mohd Salleh et al., 2017	
2		MF066633	Sumatra, Indonesia	1,013	Steiner et al., 2018	
3		MF066634	Sumatra, Indonesia	1,013	Steiner et al., 2018	
4		MF066636	Sumatra, Indonesia	1,013	Steiner et al., 2018	
5		MF066637	Sumatra, Indonesia	1,013	Steiner et al., 2018	
6		MF066638	Sumatra, Indonesia	1,013	Steiner et al., 2018	
7		MF066639	Sumatra, Indonesia	1,013	Steiner et al., 2018	
8		MF066640	Sumatra, Indonesia	1,013	Steiner et al., 2018	
9		MF066641	Malay Peninsula	1,013	Steiner et al., 2018	
10		MF066642	Malay Peninsula	1,013	Steiner et al., 2018	
11		MF066643	Malay Peninsula	1,013	Steiner et al., 2018	
12	*D. s. harrissoni*	MF066630	Sabah, Borneo, Malaysia	1,013	Steiner et al., 2018	
13		MF066631	Sabah, Borneo, Malaysia	1,013	Steiner et al., 2018	
14		MF066632	Sabah, Borneo, Malaysia	1,013	Steiner et al., 2018	
15		MF066635	Sabah, Borneo, Malaysia	1,013	Steiner et al., 2018	
16		MK909153	Sabah, Borneo, Malaysia	1,089	Margaryan et al., 2020	
17	*Rhinoceros unicornis*	MZ736693	Assam, India	1,375	Ghosh et al., 2022	
18		MZ736694	Assam, India	1,375	Ghosh et al., 2022	
19		MZ736695	Assam, India	1,375	Ghosh et al., 2022	
20		MZ736696	Assam, India	1,375	Ghosh et al., 2022	
21		MZ736697	Assam, India	1,375	Ghosh et al., 2022	
22		MZ736698	Assam, India	1,375	Ghosh et al., 2022	
23		MZ736699	Assam, India	1,375	Ghosh et al., 2022	
24		MZ736700	Assam, India	1,375	Ghosh et al., 2022	
25		MZ736701	Assam, India	1,375	Ghosh et al., 2022	
26		MZ736702	Uttar Pradesh, India	1,375	Ghosh et al., 2022	
27		MZ736703	Uttar Pradesh, India	1,375	Ghosh et al., 2022	
28		MZ736704	Bihar, India	1,375	Ghosh et al., 2022	
29		MZ736705	West Bengal, India	1,375	Ghosh et al., 2022	
30		MZ736706	West Bengal, India	1,375	Ghosh et al., 2022	
31		MZ736707	West Bengal, India	1,375	Ghosh et al., 2022	
32		MZ736708	West Bengal, India	1,375	Ghosh et al., 2022	
33		NC_001779	Assam, India	1,376	Xu et al., 1996	

No.	Species	Accession number	Location	Length (bp)	Reference	Note
34	*Rhinoceros sondaicus annamiticus*	AY739625	Vietnam	413	Fernando et al., 2006	tRNA-Pro + D-loop
35		AY739626	Java, Indonesia	413	Fernando et al., 2006	tRNA-Pro + D-loop
36		AY739627	Java, Indonesia	413	Fernando et al., 2006	tRNA-Pro + D-loop
37	*R. s. sondaicus*	AY739628	Java, Indonesia	413	Fernando et al., 2006	tRNA-Pro + D-loop
38		KY117574	Java, Indonesia	962	Mohd Salleh et al., 2017	
39		MK909146	Java, Indonesia	967	Margaryan et al., 2020	
40		AF187837	South Africa	480	Brown and Houlden, 2000	
41		AF187838	South Africa	480	Brown and Houlden, 2000	
42		AF187839	South Africa	480	Brown and Houlden, 2000	
43		AY742828	South Africa	419	Fernando et al., 2006	tRNA-Pro + D-loop
44	*Ceratotherium simum simum*	FJ004915	South Africa/ Zimbabwe	750	Coutts, 2009	
45		FJ004916	South Africa/ Namibia/ Zimbabwe	750	Coutts, 2009	
46		FJ004917	South Africa/ Namibia	750	Coutts, 2009	
47		FJ004918	Zimbabwe	750	Coutts, 2009	
48	*C. s. cottoni*	AY742829	Zaire	418	Fernando et al., 2006	tRNA-Pro + D-loop
49		FJ004919	Unknown	722	Coutts, 2009	
50		AY742830	Kenya	417	Fernando et al., 2006	tRNA-Pro + D-loop
51		AY742831	Kenya	417	Fernando et al., 2006	tRNA-Pro + D-loop
52		FJ227495	Kenya	408	Muya et al., 2011	
53	*Diceros bicornis michaeli*	KP247520	Kenya	581	Githui et al., 2017	tRNA-Pro + D-loop
54		KY472427	Kenya	477	Moodley et al., 2017	tRNA-Pro + D-loop
55		KY472532	Tanzania	477	Moodley et al., 2017	tRNA-Pro + D-loop
56		KY472538	Kenya	477	Moodley et al., 2017	tRNA-Pro + D-loop
57		AY742832	Zimbabwe	417	Fernando et al., 2006	tRNA-Pro + D-loop
58		AY742833	Zimbabwe	417	Fernando et al., 2006	tRNA-Pro + D-loop
59		KY472323	South Africa	477	Moodley et al., 2017	tRNA-Pro + D-loop
60		KY472341	South Africa	477	Moodley et al., 2017	tRNA-Pro + D-loop
61	*D. b. minor*	KY472358	Tanzania	477	Moodley et al., 2017	tRNA-Pro + D-loop
62		OK376773	South Africa	469	Stanbridge et al., 2023	
63		OK376783	South Africa	469	Stanbridge et al., 2023	
64		OK376822	South Africa	469	Stanbridge et al., 2023	
